# Simple and Accurate Exchange Energy for Density Functional Theory

**DOI:** 10.3390/molecules25153485

**Published:** 2020-07-31

**Authors:** Teepanis Chachiyo, Hathaithip Chachiyo

**Affiliations:** 1 Department of Physics, Faculty of Science, Naresuan University, Phitsanulok 65000, Thailand; 2 Thailand Center of Excellence in Physics, Ministry of Higher Education, Science, Research and Innovation, 328 Si Ayutthaya Road, Bangkok 10400, Thailand; 3 Independent Researcher, Phitsanulok 65000, Thailand; hathaithip.chachiyo@gmail.com

**Keywords:** exchange energy, density functional theory, Quantum Monte Carlo, generalized gradient approximation

## Abstract

A non-empirical exchange functional based on an interpolation between two limits of electron density, slowly varying limit and asymptotic limit, is proposed. In the slowly varying limit, we follow the study by Kleinman from 1984 which considered the response of a free-electron gas to an external periodic potential, but further assume that the perturbing potential also induces Bragg diffraction of the Fermi electrons. The interpolation function is motivated by the exact exchange functional of a hydrogen atom. Combined with our recently proposed correlation functional, tests on 56 small molecules show that, for the first-row molecules, the exchange-correlation combo predicts the total energies four times more accurately than the presently available Quantum Monte Carlo results. For the second-row molecules, errors of the core electrons exchange energies can be corrected, leading to the most accurate first- and second-row molecular total energy predictions reported to date despite minimal computational efforts. The calculated bond energies, zero point energies, and dipole moments are also presented, which do not outperform other methods.

## 1. Introduction

Total energy is a fundamental quantity in quantum mechanics as exemplified by the Schrödinger equation and the formulation of density functional theory (DFT) [1,2]. For over 90 years, however, the Schrödinger equation for atoms and molecules has never been solved analytically due to mathematical complexity of many-electron system [3]. The ability to write down the equation, but being unable to solve it, has given rise to many approximation methods such as Hartree-Fock (HF) [4], Configuration Interaction (CI) [4], Coupled Cluster (CCSD) [5], and Quantum Monte Carlo (QMC) [6]. Alternative to the wave function based methods, DFT rests firmly on a premise that total energy can be determined exactly by the density of electrons [1,2]. It breaks down the total energy into five contributions: kinetic, potential, Coulomb repulsion, exchange, and correlation. The focus of this work is on the exchange energy contribution, but the aim is the same as that of the methods aforementioned. That is, to predict the total energy as accurately as possible.

**Figure 1:**
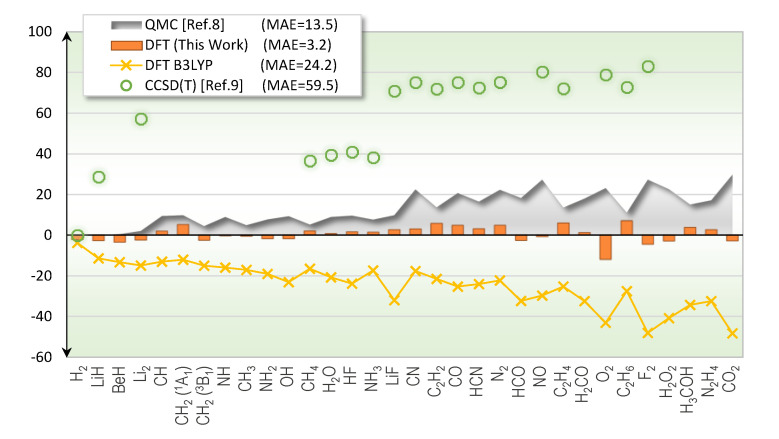
Errors of total energy predictions (kcal/mol) for first-row molecules from various theoretical methods.

Total energy of molecules can be ascertained from experiments [7], serving as a benchmark for new theoretical developments. Even though many theoretical methods are in principle exact, in practice they exhibit varying degree of errors due to limited computing power. To our knowledge, the most accurate molecular total energy benchmark reported in the literature is from the QMC method such as the one by Nemec and coworkers [8], where a set of 55 molecules were studied. Using the data from their supplementary material, we computed the mean-absolute-error (MAE) to be 13.5 kcal/mol for the first-row molecules, and 23.7 kcal/mol for the full set. The CCSD(T) benchmark using highly exhaustive computing power was also reported [9]. The MAE as compared to the experimentally derived total energies was 60 kcal/mol, which was less accurate than the QMC results as shown in Figure 1. Although there are other energetic metrics such as atomization energy typically used to address the accuracy of theoretical methods [10], we would like to initially focus on the total energy, which is at the heart of quantum mechanics both in the Schrödinger equation and in the formulation of DFT.

Over 90 years since the original idea was conceived by Thomas and Fermi, the vast developments of exchange energy functional for DFT have been extensively explored; and even though much has been understood regarding its behavior [11,12], there is plenty of room for more understanding. For example, it is well-established that the exchange energy obeys two limiting cases: (i) the slowly varying density, and (ii) the asymptotic limit where the density decays rapidly in the outer regions of molecules [13]. In 1988, Becke proposed a functional that satisfied the two limits [11]. The question remains, however, what exactly happens in the intermediate region? In this work, we explicitly use an interpolation function to merge the two limits and show that the resulting functional is simple and accurate.

Another example is related to the slowly varying limit where the exchange energy increases as a function of the reduced gradient parameter $s\equiv \left|\vec{\nabla }\rho \right|/2{\left(3\pi^{2}\right)}^{1/3}\rho^{4/3}$ [12]. Although it is well known that the exchange energy enhancement factor increases quadratically $\left(1+\mu s^{2}\right)$, the value of μ is not exactly understood [14]. An often cited value comes from a study by Kleinman which considered how free-electron eigenfunctions were perturbed by an external periodic potential [15]. The exchange energy was then evaluated and rewritten as a function of electron density and its gradient. If the wavelength of the external potential was large, it was estimated that $E_{x}=E_{x}^{LDA}-\frac{1}{2}\frac{1}{27\pi {\left(3\pi^{2}\right)}^{1/3}}\int \rho^{-4/3}{\left|\vec{\nabla }\rho \right|}^{2}d^{3}r$. This implies $\mu =\frac{8}{81}$ which is too small to give accurate predictions for atoms and molecules [14]. In this work, we assume an additional condition on the external periodic potential. That is, the potential also induces Bragg diffraction of the Fermi electrons. This assumption leads to a higher value of μ and gives accurate predictions of total energies, bond energies, zero point energies, and dipole moments of test systems, which include first- and second-row atoms and 56 small molecules.

This report is organized as follows. In Materials and Methods, we layout the mathematical form, propose the interpolating function, derive the value of μ, and fully construct the exchange functional. Then, we describe benchmarking procedures. In Results, we show that the exchange functional is accurate, particularly in comparison to the previously published Quantum Monte Carlo (QMC) calculations [8]. In Discussion, we elaborate on the significance of the results. In Conclusions, we list all the theoretical arguments that lead to the non-empirical exchange functional in this work.

While preparing the manuscript, the exchange functional has drawn interest from a few authors. It was implemented in the LibXC [16] under the name “Chachiyo exchange”. It was also used [17] to compute atomic potentials for a new initial guess method called SAP (Superposition of Atomic Potential). The study used 259 test molecules and reported that the Chachiyo exchange yielded the best initial guess wave functions on average out of hundreds functionals available in LibXC.

## 2. Results

### 2.1. Total Energy

The non-empirical exchange functional in this work is of the form

(1) $$E_{x}=\int \rho \varepsilon_{x}\frac{\;\;3x^{2}+\pi^{2}\ln\left(x+1\right)}{\left(3x+\pi^{2}\right)\ln\left(x+1\right)}d^{3}r,$$

where $x\equiv \frac{\left|\vec{\nabla }\rho \right|}{\rho^{4/3}}\frac{2}{9}{\left(\frac{\pi }{3}\right)}^{1/3}$; and $\rho \left(\vec{r}\right)$ is the total electron density. $\varepsilon_{x}$ is the Dirac exchange energy per electron for uniform electron gas [18]. To produce the results as shown in the following paragraphs, one also needs a correlation energy contribution. We use our recently published correlation functional [19]

(2) $$E_{c}=\int \rho \varepsilon_{c}{\left(1+t^{2}\right)}^{h/\varepsilon_{c}}\;d^{3}r.$$

This exchange-correlation combo was shown to give very accurate total energies for a small set of molecules. Here, we expand the size of test molecules, and the variety of atoms to cover second-row elements.

Figure 1 illustrates the accuracy of the exchange functional in this work when predicting the total energies of first-row molecules. The label “(This Work)” is from the exchange and correlation functionals in Equations (1) and (2), respectively. The exchange-correlation combo performs very well in these test molecules with the MAE of 3.2 kcal/mol, four times more accurate than the presently available QMC results. For comparison, results from the most cited DFT functional, B3LYP [20,21,22], are also shown.

**Figure 2:**
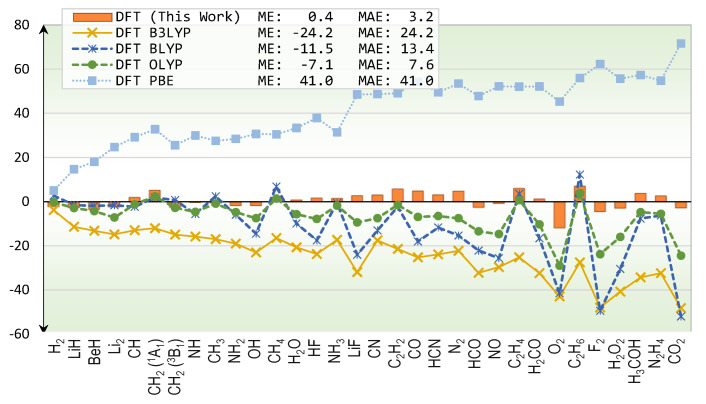
Errors of total energy predictions (kcal/mol) for first-row molecules from various DFT methods.

Results from various DFT functionals are compared in Figure 2. Comparisons between DFT’s energies and exact total energies have been reported for atoms by many authors such as Becke [21], Gill, Johnson, Frisch, and Pople [23]. Here, we simply expand the size of test systems to cover molecules. As seen from Figure 2, B3LYP functional exhibits systematic errors in that it underestimates total energies, as indicated by its negative mean-error (ME). It is interesting to see if this underestimation could be remedied by different mixing of HF exchange. The BLYP [11,22] functional, which contains zero HF mixing, still underestimates the total energies but to a lesser degree. That means simply varying the HF mixing may not fix the systematic error problems. Changing the exchange functional altogether seems to reduce this problem as shown in the OLYP [22,24]’s results, whose ME is −7.1 kcal/mol. On the other hand, the PBE [12] functional overestimates the total energies by 41.0 kcal/mol on average. The ME of our functional is 0.4 kcal/mol which could be interpreted as very small systematic error. Because the error bar of the reference total energies is about 0.6 kcal/mol, an optimistic interpretation that the functional contains no systematic error is also possible.

**Figure 3:**
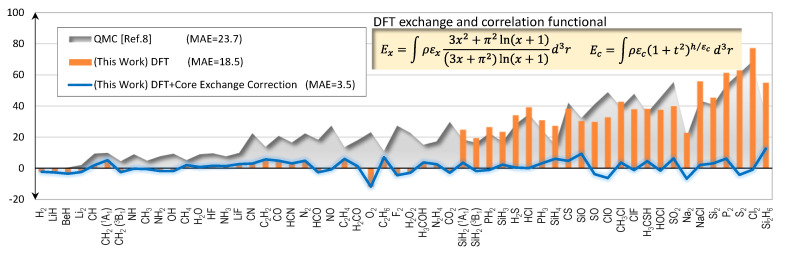
Errors of calculated total energies (kcal/mol) with second-row molecules included.

The main results of this report are the errors of the calculated total energies of 56 molecules as shown in Figure 3. The errors stay to within a few kcal/mol on average for the first-row molecules, but sharply rise to match that of the QMC for the second-row molecules. Fortunately, upon further investigation we learn that the abruptly rising error characteristics of the second-row molecules originate from the core electrons’ exchange energies. A simple ad-hoc correction scheme, as explained in Section 2.2, can be devised and used to eliminate the errors very effectively. As shown in Figure 3, after we apply the correction scheme, the errors suddenly drop; and the MAE of the full set is now only 3.5 kcal/mol.

### 2.2. Core Exchange Correction Scheme

**Figure 4:**
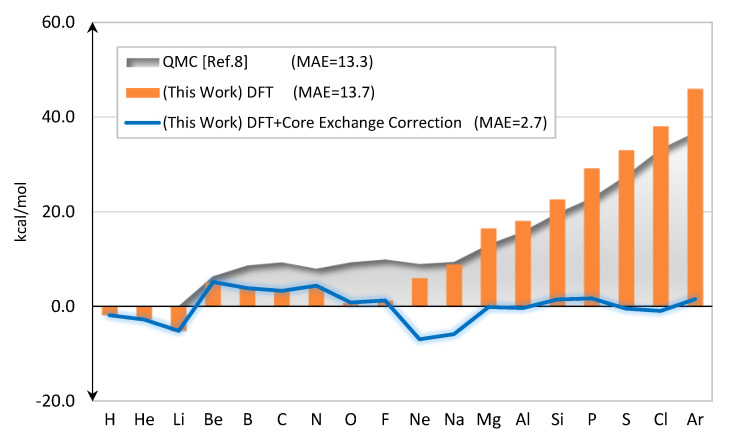
Errors of the calculated total energies as compared to the “exact” values [7] (kcal/mol). The QMC results [8] are also shown.

Figure 4 shows errors of the predicted total energies of neutral atoms. The performance is satisfactory for small atoms, but increasingly poorer starting from Ne onward. The results from QMC calculation [8] also show the same trend.

**Figure 5:**
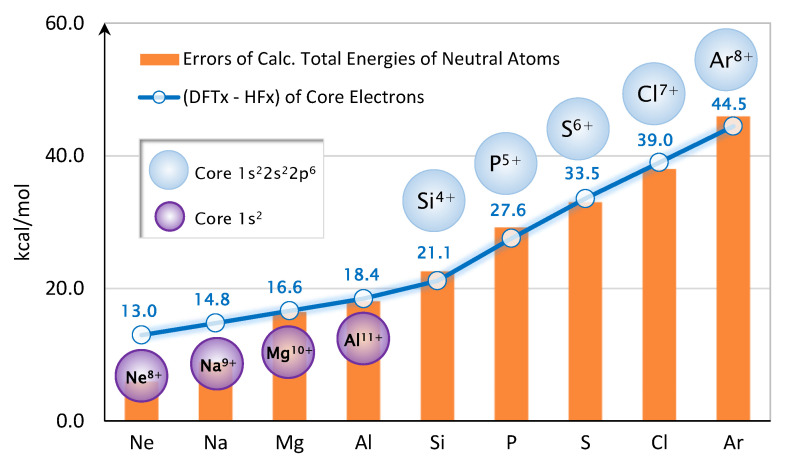
Schematic of the Core Exchange Correction.

After a few trial-and-error attempts, we discover that the errors of the predicted total energies for Ne and the second-row atoms are related to the core electrons’ exchange energy. A simple ad-hoc correction scheme for atoms and molecules can be devised very accurately. As shown in Figure 5, we first compute the error of exchange energy at Hartree-Fock (HF) density for each ion core. For example, HF calculation is performed on ${\mathrm{Ne}}^{8+}$, yielding HF orbitals and HF density. Then, the orbitals are used to compute HF exchange energy; whereas the density is used to compute the DFT exchange energy via Equation (1). The difference $\left({\mathrm{DFT}}_{x}-{\mathrm{HF}}_{x}\right)$ is reported in Figure 5.

From the graph, $\left({\mathrm{DFT}}_{x}-{\mathrm{HF}}_{x}\right)$ of the core electrons track quite accurately with the errors of the predicted total energies. Therefore, we devise an error correction scheme by simply subtracting the $\left({\mathrm{DFT}}_{x}-{\mathrm{HF}}_{x}\right)$ of the core electrons from the DFT’s total energy. In other words,

(3) $$\mathrm{Core}\;\mathrm{Exchange}\;\mathrm{Correction}\;\Delta E_{x}^{\left(\mathrm{core}\right)}\equiv -\left({\mathrm{DFT}}_{x}^{\left(\mathrm{core}\right)}-{\mathrm{HF}}_{x}^{\left(\mathrm{core}\right)}\right).$$

The corrected total energies, $E_{\mathrm{total}}^{\left(\mathrm{DFT}\right)}+\Delta E_{x}^{\left(\mathrm{core}\right)}$, are shown in Figure 4. The mean-absolute-error (MAE) is now dropped to only 2.7 kcal/mol. The values of $\left({\mathrm{DFT}}_{x}^{\left(\mathrm{core}\right)}-{\mathrm{HF}}_{x}^{\left(\mathrm{core}\right)}\right)$ are given in Figure 5. In this ad-hoc scheme, the correction is only applied to Ne through Ar.

For molecules, the correction is done “atom-wise”, and only on the second-row atoms. That is,

(4) $$\mathrm{Corrected}\;\mathrm{Molecular}\;\mathrm{Total}\;\mathrm{Energy}=E_{\mathrm{total}}^{\left(\mathrm{DFT}\right)}+\sum_{\begin{array}{c} 2\mathrm{nd}-\mathrm{row}\\ \mathrm{atoms} \end{array}}\Delta E_{x}^{\left(\mathrm{core}\right)}.$$

For example, ${\mathrm{Si}}_{2}H_{6}$ contains 2 silicon atoms. Its corrected total energy is $E_{\mathrm{total}}^{\left(\mathrm{DFT}\right)}+2\times \left(-21.1\;\mathrm{kcal}/\mathrm{mol}\right)$.

### 2.3. Bond Energy and Zero Point Energy

**Figure 6:**
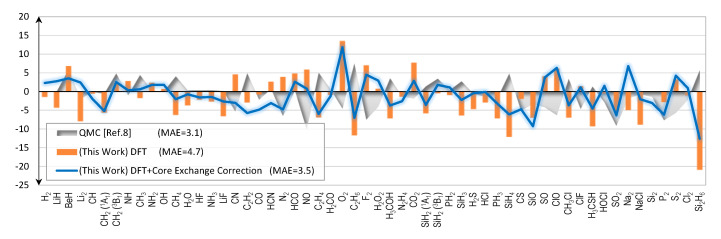
Errors of calculated bond energies (kcal/mol). The reference values are from Reference [7].

The fact that this atom-wise correction scheme works very well implies that the errors stem from the core electrons which in general do not participate in chemical bonding. If so, the DFT energies without any correction should work well when predicting the bond energies, which is the case as shown in Figure 6.

Bond energies are the atomization energies without zero point energies nor relativistic contributions. We report the bond energies to allow direct comparisons with the reported QMC results [8].

(5) $$\mathrm{Bond}\;\mathrm{Energies}=\sum_{A\in \mathrm{atoms}}E_{\mathrm{total}}^{\left(A\right)}-E_{\mathrm{total}}^{\left(M\right)}$$

The summation runs through all constituent atoms in the molecule. In Figure 6, under the label “(This Work) DFT”, errors of the predicted bond energies are shown. The molecular total energies and the atomic total energies are from the DFT calculations without any correction. The MAE is 4.7 kcal/mol.

An alternative way to compute the bond energies is to use the “exact” atomic energies, as supposed to the same method that has been used to compute the molecular energy. Typically, this will lead to huge errors if we cannot predict the molecular total energies accurately enough. It is interesting to see how the corrected molecular energies perform in this test. The results are shown in Figure 6 under the label “(This Work) DFT+Core Exchange Correction”. In this case, the molecular total energies that enter Equation (5) are the corrected ones; whereas the atomic total energies are the exact values from Reference [7]. The MAE now drops to 3.5 kcal/mol, very close to that of the QMC results. In fact, the 3.5 kcal/mol MAE for the bond energies is identical to that of the total energies. This is no coincidence as one can mathematically show that both errors are identical, provided that we use the “exact” atomic energies to evaluate the bond energies. The proof is provided in the Supplementary Material.

The accuracy of Equations (1) and (2) when predicting energy difference is less than that of the QMC method by about 1–2 kcal/mol. The performance in the atomization energy test is expected to be inferior to the CCSD(T) method which generally achieves ±1 kcal/mol or better (“chemical accuracy”) [10].

In addition to the energies previously discussed, one may look at other physical properties such as dipole moments or zero point energies. Table 1 shows the errors of calculated dipole moments which reflect the accuracy of the predicted electron density (i.e. the x-component of a dipole moment involves an integral $\int x\rho \left(\vec{r}\right)d^{3}r$). The MAE is 0.11 Debye which is comparable to that of other DFT functionals [25]. The basis set QZP-g used in this study does not contain diffuse functions which are important for dipole moment calculations. Therefore, the reported MAE may be further improved if a basis set with diffuse functions such as aug-pcseg3 [26] is used instead.

Zero point energies (ZPE) are indicators of how well the functional predicts the derivatives of energies. The first derivatives are needed for geometry optimization. At the optimized geometries, the second derivatives are needed to compute vibration frequencies; and the sum of those frequencies are then translated to the vibrational ZPEs. Due to our limited computational resources, we can only use the small 6-31G* basis set. From Table 1, the MAE is 0.12 kcal/mol which is comparable to that of the highly cited technique [27] of predicting ZPEs. Therefore, in addition to the total energies, the functionals in Equations (1) and (2) are able to predict the derivatives of energies quite accurately.

## 3. Discussion

The accuracies of Equations (1) and (2) are perplexing when considering how much total energies are involved in the calculations. Despite nearly 1,000 folds increase in total energies, 739 kcal/mol for H_2_ to more than half a million kcal/mol for Cl_2_, the errors in Figure 3 remain flat on the order of a few kcal/mol without apparent over-shooting nor under-shooting. From this data set, it is reasonable to project that even if the molecules are much larger, the errors should remain roughly the same. In other words, the DFT exchange-correlation functional combo has no systematic error. Even if the molecules contain second-row atoms, the energies can still be corrected very accurately. As evident from Figure 1, Figure 2 and Figure 3, the DFT results in this work are the most accurate first- and second-row molecular total energy predictions reported to date, representing significant advances in the field of quantum mechanics.

The success of Equations (1) and (2) does not imply that these functionals perform better than QMC method in general. QMC method has been shown to produce very accurate atomic energies with only 1.5 kcal/mol MAE [29]; but the same level of accuracy has not been achieved for molecules. QMC calculations generally require huge computing power. For example, the QMC energies shown in Figure 3 required 40 CPU hours per electron (Intel XEON, 3 GHz CPU) [8]. In contrast, our DFT calculations used 10 CPU hours to complete the full set of 56 molecules, translating to 0.01 CPU hours per electron (Intel Celeron, 1.86 GHz). Therefore, if one needs a speedy result with an overall error of a few kcal/mol, this functional combination provides a good alternative to the existing theoretical methods.

## 4. Materials and Methods

Traditionally, a dimensionless parameter s was used to quantify the inhomogeneity of electron density. The exchange energy was then written with an “enhancement factor” F(s) [12],

(6) $$E_{x}\left[\rho \right]=\int \rho \varepsilon_{x}F\left(s\right)d^{3}r.$$

Here, $\varepsilon_{x}=-\frac{3}{4}{\left(\frac{3}{\pi }\rho \right)}^{1/3}$ was the Dirac exchange energy for uniform electron density [18]. As the electron density deviated from the homogeneous case s=0, the function F(s) increased from the baseline value of 1, effectively enhancing the strength of the exchange energy from that of the uniform electron gas.

As March elaborated [30], in the asymptotic limit s→∞, the exchange energy density needed to behave as $\varepsilon_{x}F\left(s\right)\to -1/2r$. Also asymptotically, the electron density decayed exponentially $\rho \left(r\right)\to Ne^{-ar}$. This asymptotic behavior was not specific to a hydrogen atom but occurred in general [13], where the parameter a was related to the ionization potential. Therefore, to satisfy the limit, the enhancement factor was thought to take the form $F\left(s\right)=\frac{cs}{\ln s}$, which undesirably diverged to infinity at s=1. Therefore, as the first step, we propose the following modification.

(7) $$s\to \infty :\;F\left(s\right)=\frac{cs}{\ln\left(cs+1\right)}\;;\;c=\frac{4\pi }{9},$$

which is well behaved in the entire range s∈[0,∞) with an additional advantage: F(0)=1. It is easy to show that for the $\varepsilon_{x}F\left(s\right)\to -1/2r$ in this limit, the constant c has to be equal to 4π/9.

The next step is to merge F(s) in Equation (7) with the slowly varying case. It was known [14,15] that in this region, the enhancement factor grew quadratically as $1+\mu s^{2}$. Ignoring altogether the $\mu s^{2}$ behavior in the beginning and conjecturing that the quadratic dependence would emerge naturally after the interpolation, we simply try to interpolate between the *perfectly uniform* electron gas F(s)=1 in the (s=0) limit, and the $F\left(s\right)=\frac{cs}{\ln\left(cs+1\right)}$ in the asymptotic limit (s→∞) with the simplest interpolation scheme possible, namely

(8) $$F\left(s\right)=1\cdot w\left(s\right)+\frac{cs}{\ln\left(cs+1\right)}\cdot \left[1-w\left(s\right)\right].$$

Here, w(s) is the weighting function which takes a value between [0,1]. We use $w\left(s\right)=\frac{1}{ds+1}$ with the constant d controlling how rapidly the weighting function migrates from the slowly varying limit to the asymptotic limit. Substituting the w(s) into Equation (8) yields

(9) $$F\left(s\right)=\frac{\;\;dcs^{2}+\ln\left(cs+1\right)}{\left(ds+1\right)\ln\left(cs+1\right)}.$$

The choice of the weighting function $w\left(s\right)=\frac{1}{ds+1}$ is motivated by the exact exchange functional for a hydrogen atom. Consider a hydrogen atom whose normalized electron density is $\rho \left(r\right)=\frac{1}{\pi }e^{-2r}$. Recall that the electron repulsion energy $E_{e-e}=\frac{1}{2}\iint d^{3}r_{1}d^{3}r_{2}\frac{\rho \left({\vec{r}}_{1}\right)\rho \left({\vec{r}}_{2}\right)}{\left|{\vec{r}}_{1}-{\vec{r}}_{2}\right|}\;\;$ of the Kohn-Sham DFT is always there in the formalism regardless of the physical system under study. However, there is only one electron in the hydrogen atom; hence, the role of the exchange energy in this case is to cancel exactly the $E_{e-e}$ in the Kohn-Sham formalism. For a hydrogen atom,

(10) $$E_{e-e}=\int d^{3}r\rho \left(\vec{r}\right)\left[e^{-2r}\left(-\frac{1}{2}\right)+\left(1-e^{-2r}\right)\frac{1}{2r}\right]\;\;\;.$$

Therefore, the exchange energy density which cancels the above expression exactly is

(11) $$\varepsilon_{x}\left(\rho \right)F\left(s\right)=e^{-2r}\left(\frac{1}{2}\right)+\left(1-e^{-2r}\right)\left(-\frac{1}{2r}\right).$$

Notice the curious pattern which mimics an interpolation with the term $e^{-2r}$ representing the weighting function. Note that weighting function decays exponentially in r. 

Using $\rho \left(r\right)=\frac{1}{\pi }e^{-2r}$, we have $s\propto e^{2r/3}$. In other words, $\frac{1}{ds+1}$ also decays exponentially in r, approximately mimicking the behavior of the exact exchange functional of a hydrogen atom. In fact, even if we were to allow a more general form $w\left(s\right)=\frac{1}{ds^{n}+1}$, the second order Taylor expansion of F(s) in Equation (8) would have vanished for all positive integers n, except when n=1. In other words, n=1 is the only case consistent with the quadratic behavior of the enhancement factor. Therefore, we believe $w\left(s\right)=\frac{1}{ds+1}$ is the optimal form of the weighting function.

Next, we determine the value of μ in the slowly varying limit. In generalize gradient approximation, when the gradient parameter s was small, the exchange energy (per unit volume) was approximately

(12) $$\frac{E_{x}}{V}=-\frac{3}{4}{\left(\frac{3}{\pi }\right)}^{1/3}\rho^{4/3}\left(1+\mu s^{2}\right).$$

In 1984, Kleinman studied how free-electron eigenfunctions were perturbed by an external periodic potential $V_{\mathrm{ext}}\left(\vec{K}\right)e^{+i\vec{K}\cdot \vec{r}}+V_{\mathrm{ext}}\left(-\vec{K}\right)e^{-i\vec{K}\cdot \vec{r}}$ [15]. To avoid confusion, we will use the notation Equation (K-Number) when referring to a specific equation in the Kleinman’s paper. From Equation (K-17), the exchange energy per normalization volume Ω is

(13) $$\frac{E_{x}}{\Omega }=-\frac{3}{4}{\left(\frac{3}{\pi }\right)}^{1/3}\rho_{0}^{4/3}-\frac{2\pi }{3K^{2}}\left\{{\left[\rho \left(\vec{K}\right)\right]}^{2}+{\left[\rho \left(-\vec{K}\right)\right]}^{2}\right\}.$$

Using $\rho \left(\vec{K}\right)=\rho \left(-\vec{K}\right)$ as indicated in the Equation (K-10), we have

(14) $$\frac{E_{x}}{\Omega }=-\frac{3}{4}{\left(\frac{3}{\pi }\right)}^{1/3}\rho_{0}^{4/3}\left(1+\frac{4}{3}{\left(\frac{\pi }{3}\right)}^{1/3}\rho_{0}^{-4/3}\frac{2\pi }{3K^{2}}2{\left[\rho \left(\vec{K}\right)\right]}^{2}\right).$$

Comparing Equation (12) and Equation (14), we can identify

(15) $$\frac{4}{3}{\left(\frac{\pi }{3}\right)}^{1/3}\rho_{0}^{-4/3}\frac{2\pi }{3K^{2}}2{\left[\rho \left(\vec{K}\right)\right]}^{2}=\mu s^{2}=\mu \frac{1}{4{\left(3\pi^{2}\right)}^{2/3}}\frac{{\left|\vec{\nabla }\rho \right|}^{2}}{\rho^{8/3}}.$$

Using $\rho \approx \rho_{0}$ as generally the case for perturbed free-electron gas, and $k_{F}={\left(3\pi^{2}\rho_{0}\right)}^{1/3}$, we have

(16) $$\mu =\frac{32}{27}\frac{k_{F}^{4}}{K^{4}}\frac{2K^{2}{\left[\rho \left(\vec{K}\right)\right]}^{2}}{{\left|\vec{\nabla }\rho \right|}^{2}}.$$

It is possible to mathematically deduce that $\frac{2K^{2}{\left[\rho \left(\vec{K}\right)\right]}^{2}}{{\left|\vec{\nabla }\rho \right|}^{2}}=1$ on average (see Supplementary Material). Therefore,

(17) $$\mu =\frac{32}{27}{\left(\frac{k_{F}}{K}\right)}^{4}.$$

One choice of ${\left(\frac{k_{F}}{K}\right)}^{4}$ is of particular interest. Consider the case when the external potential induces Bragg diffraction of the Fermi electrons. The mathematical condition for this to occur is [31]

(18) $$2{\vec{k}}_{F}\cdot \vec{K}=K^{2}\;\mathrm{or}\;4k_{F}^{2}K^{2}\cos^{2}\left(\theta \right)=K^{4}.$$

Replacing $\cos^{2}\left(\theta \right)$ with its average value $\frac{1}{\pi }\int_{0}^{\pi }\cos^{2}\left(\theta \right)d\theta =\frac{1}{2}$, and rearranging the above expression, we have ${\left(\frac{k_{F}}{K}\right)}^{4}=\frac{1}{4}$. Hence,

(19) $$\mu =\frac{8}{27}=\frac{24}{81}.$$

Having elaborated on the choice of the weighting function, and determined the value of μ, we revisit Equation (9) and attempt to compute the constant d using Taylor expansion up to the second order:

(20) $$s\ll 1:\;F\left(s\right)\approx 1+0\cdot s+\frac{1}{2}\left(dc\right)s^{2},$$

which can readily be compared to the known $1+\mu s^{2}$ behavior of the slowly varying limit. We use the value $\mu =\frac{8}{27}$ from Equation (19), yielding

(21) $$\frac{1}{2}\left(dc\right)=\frac{8}{27}\;\mathrm{or}\;d=\frac{8}{27}\frac{2}{c}=\frac{4}{3\pi }.$$

Putting the constant d and c back into Equation (9) and defining another variable $x\equiv cs=\frac{\left|\vec{\nabla }\rho \right|}{\rho^{4/3}}\frac{2}{9}{\left(\frac{\pi }{3}\right)}^{1/3}$, we finally arrive at the non-empirical exchange functional in this work.

To allow direct comparisons with the previous QMC results [8], we use a set of 56 molecules with the exact same geometries as described in the supplementary material of Reference [8]. Experimentally derived total energies [7] and zero point energies [28] are also available for comparisons. We use a large basis set QZP [32]-g (with g GTO removed) for single point calculations, and a smaller basis 6-31G* for zero point energy calculations. The basis sets labelled “Jorge-QZP” is downloaded from the EMSL Basis Set Exchange database [33]. The reported values are calculated using the Siam Quantum program [34]. The detailed methods for solving the Kohn-Sham SCF equation for these exchange-correlation functionals are described in the supplementary material of Reference [19]. Very recently, the functionals in Equations (1) and (2) were implemented in the LibXC library [16] where the calculated energies agreed with that of the Siam Quantum’s implementation to within micro-hartrees.

After performing basis set convergence tests by comparing the basis sets TZP, QZP-g, pcseg3-g [26], and pcseg4-gh [26], we find that the pcseg3-g basis is very close to the basis set limit with an average error of only 0.2 kcal/mol. Detailed analysis of the convergence test is given in the Supplementary Material. From the test, it is reasonable to take pcseg4-gh as the basis set limit. Going from the QZP-g to pcseg4-gh basis, the total energy is lowered by about 1.4 kcal/mol. For molecules with positive errors, the lowering is good because the errors are reduced. For molecules with negative errors, the lowering is bad because the errors become even more negative. At the end, these 2 effects cancel somewhat. Because of this statistical feature, the mean-absolute-error for QZP-g and pcseg4-g are different only by 0.2 kcal/mol. Due to our limited computing power, we use QZP-g basis set for this study. The reported total energies could be about 1.4 kcal/mol above the basis set limit, and the reported MAE could be off by 0.2 kcal/mol. The basis set QZP-g should be sufficient to support the conclusion of this study.

The molecular total energies can be derived from experimental atomization energy, provided that the atomic total energies, zero-point vibrational energies, and relativistic corrections are known to high accuracies [7]. O’neill and Gill have determined non-relativistic total energies, HF energies, and correlation energies of 56 neutral molecules to within 0.6 kcal/mol (1 milli-hartree) [7], which is sufficient for our purpose. We use the non-relativistic electronic energy listed in Table 2 of Reference [7] to compute the errors of total energy predictions in this work.

## 5. Conclusions

In this study, we show that a simple and accurate exchange functional can be devised using a model that a free-electron gas is perturbed by the periodic external potential which also induces Bragg diffraction of the Fermi electrons. Using this model, we have determined the quadratic coefficient of the exchange enhancement factor to be $\mu =\frac{8}{27}$, which governs how the exchange energy is enhanced by electron density gradient in the slowly varying limit. We also show that the slowly varying limit can be merged into the asymptotic limit using an interpolation scheme, yielding the complete exchange energy functional applicable to the full range of electron density gradients. The interpolating function is $w\left(s\right)=\frac{1}{ds+1}$, which mimics the exact exchange functional of a hydrogen atom.

Tests on 56 small molecules indicate that the exchange energy functional, in combination with the recently published correlation functional, is uniquely accurate when predicting the total energies of molecules, with the MAE of only 3.2 kcal/mol for the first-row molecules. For the second-row molecules, errors of the core electron exchange energies can be corrected, leading to the most accurate molecular total energy predictions reported to date despite minimal computational efforts.

## Figures and Tables

**Table 1:** Errors of the calculated total energies, bond energies, dipole moments (Debye), and zero point energies of test molecules. All energies are in kcal/mol. Experimental dipole moments and zero point energies are from Reference [28].

	Equations (1) and (2)	Equations (1) and (2)$+\Delta E_{x}^{\left(\mathrm{core}\right)}$
**Total Energy**		
ME	16.9	1.0
MAE	18.5	3.5
first-row ME	0.4	0.4
first-row MAE	3.2	3.2
**Bond Energy**		
ME	−1.9	−1.0
MAE	4.7	3.5
first-row ME	−0.3	−0.4
first-row MAE	4.4	3.2
**Dipole Moment**		
MAE	0.11	
**Zero Point Energy**		
MAE (6–31G*)	0.12	

## Supplementary Materials

The following are available online, Table S1: Atomic total energies, Table S2: 56 molecular total energies, Table S3: DFT total energies of the first-row molecules, Table S4: Errors of the predicted atomic total energies, Table S5: Errors of the predicted molecular total energies, Table S6: Error of the predicted DFT and CCSD(T) total energies, Table S7: Calculated dipole moments and zero point energies, additional theoretical proofs, and basis set convergence tests. Output files from Siam Quantum software are also available.
