# Supplementary material for: Simple and Accurate Exchange Energy for Density Functional Theory
Source: Molecules. 2020 Jul 31;25(15):3485. doi: 10.3390/molecules25153485 (PMC7436057; doi:10.3390/molecules25153485)
Supplement: Supplementary file 1 [file molecules-25-03485-s001.zip › supplementary_material.pdf]

Supplementary Material

Simple and accurate exchange energy for density functional theory

Teepanis Chachiyo,[1,2,*] and Hathaithip Chachiyo[3]

[1] Department of Physics, Faculty of Science, Naresuan University, Phitsanulok 65000 Thailand
email: teepanisc@nu.ac.th

[2] Thailand Center of Excellence in Physics, Ministry of Higher Education, Science, Research and Innovation, 328 Si Ayutthaya Road, Bangkok 10400, Thailand

[3] Independent Researcher, Phitsanulok 65000, Thailand   email: hathaithip.chachiyo@gmail.com

[*] Corresponding author

Acknowledgements

Support by Thailand Center of Excellence in Physics grant No. ThEP-60-PET-NU9 is gratefully acknowledged.

Page

1) Proof that $\frac{2K^2[\rho(\vec{K})]^2}{|\vec{\nabla}\rho|^2} = 1$ on average S2

2) Proof that the errors of total energies and bond energies are the same (magnitude) S3

3) Calculated Energies S5

4) Errors of the Calculated Energies S8

5) Dipole Moments and Zero Point Energies S11

6) Molecular Coordinates S12

7) Basis Set Convergence Test S13

8) References S16

Proof that $\frac{2K^2[\rho(\vec{K})]^2}{|\vec{\nabla}\rho|^2} = 1$ on average

Upon perturbation by a periodic external potential $V_{\mathrm{ext}}(\vec{K})e^{+i\vec{K}\cdot\vec{r}} + V_{\mathrm{ext}}(-\vec{K})e^{-i\vec{K}\cdot\vec{r}}$, the free-electron gas density becomes

$$\rho = \rho_0 + \rho(\vec{K})e^{+i\vec{K}\cdot\vec{r}} + \rho(-\vec{K})e^{-i\vec{K}\cdot\vec{r}} = \rho_0 + 2\rho(\vec{K})\cos(\vec{K}\cdot\vec{r}), \tag{S1}$$

which leads to the gradient of electron density

$$\vec{\nabla}\rho = -2\rho(\vec{K})\sin(\vec{K}\cdot\vec{r})\vec{K} \tag{S2}$$

and

$$|\vec{\nabla}\rho|^2 = 4K^2[\rho(\vec{K})]^2\sin^2(\vec{K}\cdot\vec{r}). \tag{S3}$$

Therefore, the average of $|\vec{\nabla}\rho|^2$ over the normalization volume Ω is

$$\frac{1}{\Omega}\int|\vec{\nabla}\rho|^2 d^3r = \frac{1}{\Omega}4[\rho(\vec{K})]^2 K^2 \int \sin^2(\vec{K}\cdot\vec{r})d^3r. \tag{S4}$$

Since the volume is periodic, replacing the integrand with $\cos^2(\vec{K}\cdot\vec{r})$ would only shift the phase, but the integrated value would not change. In other words,

$$\int \sin^2(\vec{K}\cdot\vec{r})d^3r = \int \cos^2(\vec{K}\cdot\vec{r})d^3r = \frac{1}{2}\int\left[\cos^2(\vec{K}\cdot\vec{r}) + \sin^2(\vec{K}\cdot\vec{r})\right]d^3r = \frac{\Omega}{2}. \tag{S5}$$

Hence,

$$\frac{1}{\Omega}\int\left|\vec{\nabla}\rho\right|^2 d^3r = 2K^2\left[\rho\left(\vec{K}\right)\right]^2. \tag{S6}$$

Consider $\frac{2K^2\left[\rho\left(\vec{K}\right)\right]^2}{\left|\vec{\nabla}\rho\right|^2}$. If we replace the term $\left|\vec{\nabla}\rho\right|^2$ by its average value as shown in the above expression, we have

$$\frac{2K^2\left[\rho\left(\vec{K}\right)\right]^2}{\left|\vec{\nabla}\rho\right|^2} = 1 \textit{ on average}. \tag{S7}$$

Proof that the errors of total energies and bond energies are the same (magnitude)

Let $E_{\text{exact}}^{(\text{M})}$ and $E_{\text{exact}}^{(\text{A})}$ be the exact total energies of molecules and of atoms, respectively. If a theory predicts the total energy of a molecule to be $E_{\text{theory}}^{(\text{M})}$, then the error is equal to

$$\Delta E^{(\text{M})} = E_{\text{theory}}^{(\text{M})} - E_{\text{exact}}^{(\text{M})}. \tag{S8}$$

A (positive value) bond energy is computed by subtracting the molecular total energy from the sum of atomic energies.

$$E_{\text{exact}}^{(\text{Bond})} = \sum_{\text{A}\in\text{atoms}} E_{\text{exact}}^{(\text{A})} - E_{\text{exact}}^{(\text{M})}. \tag{S9}$$

For a theoretical prediction of bond energy, both the molecular energy and the atomic energies are from the same theory, relying on a certain degree of error cancellation, even though the $E_{\text{theory}}^{(\text{M})}$ and $E_{\text{theory}}^{(\text{A})}$ are both off by huge amount, the difference is still quite accurate.

However, let us evaluate the bond energy using

$$E_{\text{theory}}^{(\text{Bond})} = \sum_{\text{A}\in\text{atoms}} E_{\text{exact}}^{(\text{A})} - E_{\text{theory}}^{(\text{M})}. \tag{S10}$$

Notice how we are using the exact atomic energies instead of the theoretical ones. The error of the bond energy in Eq. (S10) is

$$\begin{aligned} \Delta E^{(\text{Bond})} &= E_{\text{theory}}^{(\text{Bond})} - E_{\text{exact}}^{(\text{Bond})} \\ &= \left(\sum_{\text{A}\in\text{atoms}} E_{\text{exact}}^{(\text{A})} - E_{\text{theory}}^{(\text{M})}\right) - E_{\text{exact}}^{(\text{Bond})}. \end{aligned} \tag{S11}$$

Substituting $E_{\text{exact}}^{(\text{Bond})} = \sum_{\text{A}\in\text{atoms}} E_{\text{exact}}^{(\text{A})} - E_{\text{exact}}^{(\text{M})}$ from Eq. (S9) into the above expression yields,

$$\Delta E^{(\text{Bond})} = \left(\sum_{\text{A}\in\text{atoms}} E_{\text{exact}}^{(\text{A})} - E_{\text{theory}}^{(\text{M})}\right) - \left(\sum_{\text{A}\in\text{atoms}} E_{\text{exact}}^{(\text{A})} - E_{\text{exact}}^{(\text{M})}\right) \tag{S12}$$

The atomics energies cancel and we are left with

$$\Delta E^{(\text{Bond})} = -E_{\text{theory}}^{(\text{M})} + E_{\text{exact}}^{(\text{M})}, \tag{S13}$$

which is precisely the negative of Eq. (S8). Therefore,

$$\Delta E^{(\text{M})} = -\Delta E^{(\text{Bond})} \tag{S14}$$

In other words, the error of predicted the molecular total energies is of the same magnitude as that of the bond energies prediction, provided that we use the exact atomic energies as in Eq. (S10).

Calculated Energies

Reference total energies used to compute the errors are from Ref. [1].

Table S1. Atomic total energies

Color Code
Calculated Values << Copied by values from Siam Quantum
Analysis << Worksheet formula

		---- Core Exchange Correction Scheme for Ne and 2nd-row atoms -----					
	(This Work) Etotal QZP-gh [Ha]	Exchange Energy @HF Density				(This Work)	
name		Core Config.	DFT ex.	HF ex.	Correction [Ha]	Corected Energies [Ha]	
H	-0.502981				0	-0.502981	(No Correction)
He	-2.908144				0	-2.908144	(No Correction)
Li	-7.486352				0	-7.486352	(No Correction)
Be	-14.659224				0	-14.659224	(No Correction)
B	-24.647743				0	-24.647743	(No Correction)
C	-37.839799				0	-37.839799	(No Correction)
N	-54.582274				0	-54.582274	(No Correction)
O	-75.066050				0	-75.066050	(No Correction)
F	-99.731913				0	-99.731913	(No Correction)
Ne	-128.928050	Ne8+ [1s]	-6.0070	-6.0277	-0.0207	-128.948712	
Na	-162.240410	Na9+ [1s]	-6.6291	-6.6526	-0.0235	-162.263942	
Mg	-200.026734	Mg10+[1s]	-7.2511	-7.2776	-0.0265	-200.053214	
Al	-242.317156	Al11+[1s]	-7.8733	-7.9027	-0.0294	-242.346556	
Si	-289.322973	Si4+[1s2s2p]	-19.1780	-19.2117	-0.0337	-289.356653	
P	-341.212425	P5+[1s2s2p]	-20.9280	-20.9719	-0.0439	-341.256335	
S	-398.057341	S6+[1s2s2p]	-22.6763	-22.7297	-0.0535	-398.110801	
Cl	-460.087376	Cl7+[1s2s2p]	-24.4236	-24.4858	-0.0621	-460.149526	
Ar	-527.466732	Ar8+[1s2s2p]	-26.1695	-26.2403	-0.0709	-527.537593	

Table S2. 56 molecular total energies

name	(This Work) Etotal QZP-gh [Ha]	(This Work) Corected Etot QZP-gh [Ha]	(This Work) Ebond QZP-gh [kcal/mol]	(This Work) Corrected Ebond QZP-gh [kcal/mol]
H2	-1.178148	-1.178148	108.04833	111.789543
LiH	-8.074777	-8.074777	53.616912	60.66572539
BeH	-15.252419	-15.252419	56.610132	53.35022091
Li2	-14.998953	-14.998953	16.471494	26.82790899
CH	-38.475660	-38.475660	83.383448	81.99037703
CH2 (1A1)	-39.126332	-39.126332	176.06094	176.538472
CH2 (3B1)	-39.152413	-39.152413	192.42701	192.9045444
NH	-55.223226	-55.223226	86.578098	84.10257364
CH3	-39.836455	-39.836455	306.04449	308.3926278
NH2	-55.882231	-55.882231	184.48462	183.8797044
OH	-75.739939	-75.739939	107.24637	108.3325938
CH4	-40.512545	-40.512545	414.67201	418.8907564
H2O	-76.437104	-76.437104	229.09866	232.0554828
HF	-100.456684	-100.456684	139.17531	139.7990522
NH3	-56.562424	-56.562424	295.68682	296.952504
LiF	-107.430160	-107.430160	132.9661	136.8974487
CN	-92.720175	-92.720175	187.0618	179.4519981
C2H2	-77.326369	-77.326369	402.11367	399.3275236
CO	-113.318379	-113.318379	258.86645	254.818386
HCN	-93.426175	-93.426175	314.45813	308.7189326
N2	-109.534502	-109.534502	232.14961	223.4573492
HCO	-113.861188	-113.861188	283.85889	281.6814355
NO	-129.905881	-129.905881	161.61944	156.4889194
C2H4	-78.579291	-78.579291	557.08338	558.0384538
H2CO	-114.507057	-114.507057	373.52245	373.2156029
O2	-150.345597	-150.345597	133.97137	132.402599
C2H6	-79.816232	-79.816232	702.02488	706.7211564
F2	-199.537481	-199.537481	46.219204	43.72548187
H2O2	-151.568159	-151.568159	269.88991	272.0623441
H3COH	-115.724642	-115.724642	506.31787	509.7522337
N2H4	-111.873459	-111.873459	437.36589	436.1560498
CO2	-188.605665	-188.605665	397.69412	392.8616669
SiH2 (1A1)	-290.562529	-290.596209	146.58243	148.8508751
SiH2 (3B1)	-290.538366	-290.572046	131.41992	133.6883657
PH2	-342.461371	-342.505281	152.47474	154.5436403
SiH3	-291.184418	-291.218098	221.19832	225.3373673
H2S	-399.348422	-399.401882	178.91485	183.1586961
HCl	-460.756891	-460.819041	104.50165	107.3298337
PH3	-343.097163	-343.141073	235.81489	239.7543955
SiH4	-291.831002	-291.864682	311.31055	317.3202039
CS	-436.168105	-436.221565	170.03308	167.2720408
SiO	-364.685259	-364.718939	185.89087	183.6337207
SO	-473.330888	-473.384348	130.20632	129.9245644
ClO	-535.267984	-535.330134	71.886221	72.05941341
CH3Cl	-500.056311	-500.118461	389.17693	392.4826512
ClF	-559.921683	-559.983833	64.253197	63.96391475
H3CSH	-438.651663	-438.705123	465.98785	470.7092269
HOCl	-535.919752	-535.981902	165.25146	167.2952587
SO2	-548.595256	-548.648716	254.65272	253.5865851
Na2	-324.499718	-324.546782	11.858672	23.58305793
NaCl	-622.471571	-622.557253	90.226438	97.04620985
Si2	-578.766347	-578.833707	75.552758	72.6072291
P2	-682.606650	-682.694470	114.08121	110.7365822
S2	-796.284027	-796.390947	106.26558	107.2708479
Cl2	-920.267031	-920.391331	57.905939	59.82109775
Si2H6	-582.479772	-582.547132	512.01001	520.2881164

Table S3. DFT total energies of the first-row molecules.

Name	Siam Quantum B3LYP/QZP-g [Hartrees]	Siam Quantum BLYP/QZP-g [Hartrees]	Siam Quantum OLYP/QZP-g [Hartrees]	Siam Quantum PBE/QZP-g [Hartrees]
H2	-1.18056	-1.17014	-1.17496	-1.16654
LiH	-8.08863	-8.07295	-8.07506	-8.04712
BeH	-15.26792	-15.24996	-15.25369	-15.21809
Li2	-15.01879	-14.99773	-15.00665	-14.95573
CH	-38.49950	-38.48258	-38.48077	-38.43237
CH2 (1A1)	-39.15382	-39.13167	-39.13090	-39.08233
CH2 (3B1)	-39.17228	-39.14730	-39.15291	-39.10773
NH	-55.24805	-55.23170	-55.23017	-55.17488
CH3	-39.86269	-39.83175	-39.83695	-39.79174
NH2	-55.90976	-55.88897	-55.88703	-55.83415
OH	-75.77387	-75.76029	-75.74915	-75.68823
CH4	-40.54208	-40.50500	-40.51359	-40.46725
H2O	-76.47128	-76.45418	-76.44742	-76.38508
HF	-100.49716	-100.48725	-100.47177	-100.39879
NH3	-56.59238	-56.56566	-56.56763	-56.51471
LiF	-107.48529	-107.47279	-107.44945	-107.35712
CN	-92.75304	-92.74576	-92.73699	-92.64741
C2H2	-77.36979	-77.33936	-77.33786	-77.25720
CO	-113.36633	-113.35492	-113.33719	-113.23967
HCN	-93.46932	-93.44985	-93.44160	-93.35214
N2	-109.57759	-109.56654	-109.55413	-109.45692
HCO	-113.90849	-113.89237	-113.87850	-113.78081
NO	-129.95206	-129.94560	-129.92820	-129.82142
C2H4	-78.62906	-78.58318	-78.58777	-78.50575
H2CO	-114.56070	-114.53550	-114.52547	-114.42588
O2	-150.39529	-150.39304	-150.37298	-150.25452
C2H6	-79.87133	-79.80794	-79.82133	-79.73820
F2	-199.60687	-199.60916	-199.56833	-199.43100
H2O2	-151.62851	-151.61218	-151.58899	-151.47478
H3COH	-115.78533	-115.74284	-115.73822	-115.63915
N2H4	-111.92927	-111.88770	-111.88641	-111.79023
CO2	-188.67806	-188.68408	-188.64015	-188.48690

Errors of the Calculated Energies

Refer to the supplemental material of Ref. [2] for the reported QMC total energies and bond energies. We calculated the errors as compared to the experimentally derived energies from Ref. [1].

Table S4. Errors of the predicted atomic total energies.

	Error of Etot		
	As IS	Corrected	DMC
	QZP-gh [kcal/mol]	QZP-gh [kcal/mol]	Nemec2010 [kcal/mol]
**name**			
H	-1.9	-1.9	0.0
He	-2.8	-2.8	0.0
Li	-5.2	-5.2	0.1
Be	5.1	5.1	6.3
B	3.9	3.9	8.6
C	3.3	3.3	9.3
N	4.3	4.3	7.9
O	0.8	0.8	9.3
F	1.2	1.2	9.9
Ne	6.0	-7.0	8.9
Na	8.9	-5.9	9.4
Mg	16.5	-0.1	13.1
Al	18.1	-0.3	15.8
Si	22.6	1.5	19.7
P	29.2	1.7	22.9
S	33.0	-0.5	27.7
Cl	38.0	-1.0	33.3
Ar	46.0	1.5	36.8
**Average**	12.6	-0.1	13.3
**MAE**	13.7	2.7	13.3
**First row MAE**	3.4	3.5	6.0
**First row Avg**	1.5	0.2	6.0

Table S5. Errors of the predicted molecular total energies.

	Error of Etot			Error of E_bond		
	As IS	Corrected	DMC	As IS	Corrected	DMC
	**QZP-gh [kcal/mol]**	**QZP-gh [kcal/mol]**	**Nemec2010 [kcal/mol]**	**QZP-gh [kcal/mol]**	**QZP-gh [kcal/mol]**	**Nemec2010 [kcal/mol]**
**name**						
H2	-2.3	-2.3		-1.5	2.3	
LiH	-2.7	-2.7	0.0	-4.3	2.7	0.0
BeH	-3.5	-3.5	0.5	6.8	3.5	5.8
Li2	-2.4	-2.4	2.1	-7.9	2.4	-2.0
CH	2.0	2.0	9.4	-0.6	-2.0	-0.1
CH2 (1A1)	5.2	5.2	9.8	-5.7	-5.2	-0.5
CH2 (3B1)	-2.5	-2.5	4.5	2.0	2.5	4.8
NH	-0.3	-0.3	8.9	2.8	0.3	-1.0
CH3	-0.6	-0.6	4.9	-1.7	0.6	4.4
NH2	-1.8	-1.8	7.7	2.4	1.8	0.2
OH	-1.8	-1.8	9.3	0.7	1.8	0.0
CH4	2.0	2.0	5.2	-6.3	-2.0	4.1
H2O	0.8	0.8	9.0	-3.7	-0.8	0.3
HF	1.6	1.6	9.6	-2.2	-1.6	0.3
NH3	1.4	1.4	7.6	-2.7	-1.4	0.3
LiF	2.7	2.7	9.8	-6.6	-2.7	0.1
CN	3.0	3.0	22.5	4.6	-3.0	-5.3
C2H2	5.7	5.7	13.7	-2.9	-5.7	4.9
CO	4.8	4.8	20.8	-0.8	-4.8	-2.2
HCN	3.1	3.1	16.5	2.6	-3.1	0.7
N2	4.8	4.8	22.4	3.9	-4.8	-6.5
HCO	-2.6	-2.6	18.3	4.8	2.6	0.3
NO	-0.7	-0.7	27.3	5.9	0.7	-10.1
C2H4	6.0	6.0	13.5	-6.9	-6.0	5.0
H2CO	1.2	1.2	18.0	-0.9	-1.2	0.6
O2	-11.9	-11.9	23.1	13.4	11.9	-4.5
C2H6	7.0	7.0	11.1	-11.7	-7.0	7.5
F2	-4.5	-4.5	27.3	7.0	4.5	-7.5
H2O2	-2.9	-2.9	22.6	0.8	2.9	-4.0
H3COH	3.7	3.7	15.0	-7.2	-3.7	3.6
N2H4	2.6	2.6	17.2	-1.4	-2.6	-1.3
CO2	-2.9	-2.9	29.7	7.7	2.9	-1.9
SiH2 (1A1)	24.7	3.6	18.4	-5.8	-3.6	1.4
SiH2 (3B1)	19.3	-1.8	16.3	-0.5	1.8	3.4
PH2	26.4	-1.1	22.5	-1.0	1.1	0.4
SiH3	23.4	2.3	17.0	-6.4	-2.3	2.7
H2S	34.0	0.5	28.3	-4.7	-0.5	-0.6
HCl	39.1	0.1	34.7	-2.9	-0.1	-1.4
PH3	30.8	3.2	24.7	-7.2	-3.2	-1.8
SiH4	27.2	6.1	14.9	-12.1	-6.1	4.8
CS	38.3	4.7	42.1	-2.0	-4.7	-5.1
SiO	30.4	9.3	32.3	-7.0	-9.3	-3.3
SO	29.8	-3.8	41.2	4.1	3.8	-4.2
ClO	32.7	-6.3	48.9	6.1	6.3	-6.3
CH3Cl	42.7	3.7	39.2	-7.0	-3.7	3.4
ClF	37.8	-1.2	47.9	1.4	1.2	-4.6
H3CSH	38.1	4.6	35.7	-9.3	-4.6	1.3
HOCl	37.5	-1.5	45.5	-0.5	1.5	-2.9
SO2	39.9	6.3	55.5	-5.3	-6.3	-9.1
Na2	22.8	-6.8	20.6	-5.0	6.8	-1.8
NaCl	55.8	2.0	43.4	-8.9	-2.0	-0.7
Si2	45.3	3.1	40.8	-0.1	-3.1	-1.4
P2	61.3	6.2	53.5	-2.8	-6.2	-7.7
S2	62.9	-4.2	61.1	3.2	4.2	-5.7
Cl2	77.1	-0.9	68.8	-1.0	0.9	-2.2
Si2H6	54.9	12.6	33.7	-20.9	-12.6	5.7
**Average**	16.9	1.0	23.7	-1.9	-1.0	-0.7
**MAE**	18.5	3.5	23.7	4.7	3.5	3.1
**first-row AVG**	0.4	0.4	13.5	-0.3	-0.4	-0.1
**first-row MAE**	3.2	3.2	13.5	4.4	3.2	2.9
**second-row AVG**	38.8	1.7	37.0	-4.0	-1.7	-1.5
**second-row MAE**	38.8	4.0	37.0	5.2	4.0	3.4

3) Other QMC results. Refer to Ref. [3] for the reported QMC total energies. Here we computed the errors in kcal/mol as compared to the exact energies (quoted in the references).

	QMC Type		[Brown2007] QMC - exact [kcal/mol]
Li	DMC2-MDBF		0.00
Be	DMC2-MDBF		0.03
B	DMC2-MDBF		0.21
C	DMC2-MDBF		0.72
N	DMC2-MDBF		1.19
O	DMC2-MDBF		3.51
F	DMC2-MDBF		4.08
Ne	DMC2-MDBF		2.32
		**MAE**	1.51

4) CCSD(T) and DFT results. Refer to Table II of Ref. [4] for the reported CCSD total energy predictions. The DFT/QZP-g results are from Siam Quantum software using the same geometries as in the QMC calculations [2]. The exact energies are from Ref. [1].

Table S6. Error of the predicted DFT and CCSD(T) total energies.

Name	[Ranasinghe 2013] CCSD-Exact [kcal/mol]	B3LYP-Exact [kcal/mol]	BLYP-Exact [kcal/mol]	OLYP-Exact [kcal/mol]	PBE-Exact [kcal/mol]	This Work -Exact [kcal/mol]
H2	0.0	-3.8	2.7	-0.3	5.0	-2.3
LiH	28.7	-11.4	-1.6	-2.9	14.6	-2.7
BeH		-13.3	-2.0	-4.3	18.0	-3.5
Li2	57.3	-14.9	-1.6	-7.2	24.7	-2.4
CH		-13.0	-2.4	-1.2	29.1	2.0
CH2 (1A1)		-12.1	1.8	2.3	32.8	5.2
CH2 (3B1)		-15.0	0.7	-2.8	25.5	-2.5
NH		-15.9	-5.6	-4.7	30.0	-0.3
CH3		-17.1	2.4	-0.9	27.5	-0.6
NH2		-19.0	-6.0	-4.8	28.4	-1.8
OH		-23.1	-14.5	-7.6	30.7	-1.8
CH4	36.7	-16.5	6.8	1.4	30.5	2.0
H2O	39.5	-20.7	-10.0	-5.7	33.4	0.8
HF	41.0	-23.8	-17.6	-7.9	37.9	1.6
NH3	38.3	-17.4	-0.6	-1.8	31.4	1.4
LiF	71.0	-31.9	-24.1	-9.4	48.5	2.7
CN	75.3	-17.6	-13.0	-7.5	48.7	3.0
C2H2	72.0	-21.5	-2.4	-1.5	49.1	5.7
CO	75.2	-25.2	-18.1	-7.0	54.2	4.8
HCN	72.5	-24.0	-11.8	-6.6	49.6	3.1
N2	75.3	-22.3	-15.3	-7.6	53.4	4.8
HCO		-32.3	-22.2	-13.5	47.8	-2.6
NO	80.4	-29.7	-25.7	-14.7	52.3	-0.7
C2H4	72.2	-25.3	3.5	0.6	52.1	6.0
H2CO		-32.4	-16.6	-10.3	52.2	1.2
O2	79.0	-43.0	-41.6	-29.0	45.3	-11.9
C2H6	72.8	-27.6	12.2	3.8	56.0	7.0
F2	83.1	-48.0	-49.5	-23.9	62.3	-4.5
H2O2		-40.8	-30.5	-16.0	55.7	-2.9
H3COH		-34.3	-7.7	-4.8	57.4	3.7
N2H4		-32.4	-6.3	-5.5	54.8	2.6
CO2		-48.3	-52.1	-24.5	71.7	-2.9
**COUNT**	18	32	32	32	32	32
**MAE**	59.5	24.2	13.4	7.6	41.0	3.2
**ME**	59.5	-24.2	-11.5	-7.1	41.0	0.4

Dipole Moments and Zero Point Energies

Table S7. Calculated dipole moments and zero point energies.

	Dipole			ZPE			
	(This Work) QZP-gh Calc. [Debye]		(This Work) QZP-gh Calc. - exp [Debye]	exp. ZPE [kcal/mol]	----------- This Work --------- 6-31G* ZPE [Ha]	6-31G* ZPE [kcal/mol]	6-31G* Calc. - exp [kcal/mol]
**name**							
H2	0.0000			6.2	0.00996	6.2	0.0
LiH	5.7155		-0.1685	2.0	0.00307	1.9	-0.1
BeH	0.2744			2.9	0.00455	2.9	-0.1
Li2	0.0000			0.5	0.00073	0.5	0.0
CH	1.4316		-0.0284	4.0	0.00614	3.9	-0.2
CH2 (1A1)	1.7713				0.01600	10.0	
CH2 (3B1)	0.5995				0.01705	10.7	
NH	1.4964		0.1064	4.6	0.00716	4.5	-0.1
CH3	0.0000				0.02916	18.3	
NH2	1.7602			11.5	0.01844	11.6	0.1
OH	1.6224		-0.0326	5.3	0.00807	5.1	-0.2
CH4	0.0000			27.1	0.04417	27.7	0.6
H2O	1.8436		-0.0110	12.9	0.02065	13.0	0.1
HF	1.7639		-0.0623	5.9	0.00884	5.5	-0.3
NH3	1.5085		0.0367	20.6	0.03373	21.2	0.5
LiF	6.0996		-0.2278	1.3	0.00218	1.4	0.1
CN	1.1573			2.9	0.00475	3.0	0.0
C2H2	0.0002			15.3	0.02459	15.4	0.1
CO	0.1849		0.0751	3.1	0.00481	3.0	-0.1
HCN	2.9238		-0.0614	8.7	0.01422	8.9	0.2
N2	0.0006			3.4	0.00535	3.4	0.0
HCO	1.3894			7.8	0.01259	7.9	0.1
NO	0.2064		0.0477	2.7	0.00431	2.7	0.0
C2H4	0.0000				0.04981	31.3	
H2CO	2.2030		-0.1290	16.1	0.02588	16.2	0.1
O2	0.0002			2.3	0.00356	2.2	0.0
C2H6	0.0000				0.07341	46.1	
F2	0.0000			1.3	0.00228	1.4	0.1
H2O2	1.7142		0.1412		0.02535	15.9	
H3COH	1.5683		-0.1317		0.05006	31.4	
N2H4	1.8773		0.1273		0.05197	32.6	
CO2	0.0002			6.2	0.00979	6.1	-0.1
SiH2 (1A1)	0.2229			7.2	0.01132	7.1	-0.1
SiH2 (3B1)	0.0490				0.01180	7.4	
PH2	0.6047				0.01310	8.2	
SiH3	0.0239				0.02069	13.0	
H2S	1.0313		0.0530	9.2	0.01479	9.3	0.1
HCl	1.1216		0.0130	4.2	0.00657	4.1	-0.1
PH3	0.6647		0.0907	14.6	0.02354	14.8	0.2
SiH4	0.0000			19.2	0.03035	19.0	-0.2
CS	1.9319		-0.0261	1.8	0.00282	1.8	-0.1
SiO	2.8138		-0.2844	1.8	0.00269	1.7	-0.1
SO	1.3671		-0.1829	1.6	0.00243	1.5	-0.1
ClO	1.3514		0.0544	1.2	0.00187	1.2	0.0
CH3Cl	1.8790		-0.0173		0.03717	23.3	
ClF	0.7458		-0.1423	1.1	0.00170	1.1	-0.1
H3CSH	1.5600		0.0400		0.04523	28.4	
HOCl	1.5324		0.2324	8.0	0.01263	7.9	0.0
SO2	1.5061		-0.1270	4.3	0.00639	4.0	-0.3
Na2	0.0000			0.2	0.00034	0.2	0.0
NaCl	8.6939		-0.3073	0.5	0.00078	0.5	0.0
Si2	0.0000			0.7	0.00121	0.8	0.0
P2	0.0007			1.1	0.00175	1.1	0.0
S2	0.0002			1.0	0.00154	1.0	-0.1
Cl2	0.0000			0.8	0.00115	0.7	-0.1
Si2H6	0.0000				0.04776	30.0	
		**Avg E**	-0.03			**Avg E**	-0.003
		**MAE**	0.11			**MAE**	0.120
		**Count**	28			**Count**	42

For dipole moments, refer to the experimental values from Ref. [5]. For the zero point energies, we calculated the “exp.” values listed above from experimental vibration energies in Ref. [5]. For diatomic molecules where the anharmonic term $\omega_e x_e$ was available, we used

$$\mathrm{ZPE} = \frac{1}{2}\omega_e - \frac{1}{4}\omega_e x_e. \tag{S15}$$

For polyatomic molecules, we used

$$\mathrm{ZPE} = \sum_{\text{all modes}} \frac{1}{2}\omega_e. \tag{S16}$$

Molecular Coordinates

For H2 we used experimental bond length of 0.741 angstroms. For other 55 molecules, the geometries could be downloaded from the supplementary material of Ref. [2], (doi: 10.1063/1.3288054)

Basis Set Convergence Test

We test the basis set convergence using the basis TZP, QZP-g, pcseg3-g[6], and pcseg4-gh[6], on the order of the basis size. Due to our limited computing resources, the test set only includes 15 first-row diatomic molecules.

Using the exchange-correlation functional in this work, the calculated total energies are shown below.

Name	Siam Quantum TZP [Hartrees]	Siam Quantum QZP-g [Hartrees]	Siam Quantum pcseg3-g [Hartrees]	Siam Quantum pcseg4-gh [Hartrees]
H2	-1.177388	-1.178148	-1.178281	-1.178298
LiH	-8.073748	-8.074777	-8.074882	-8.074975
BeH	-15.251245	-15.252419	-15.252565	-15.252752
Li2	-14.998035	-14.998953	-14.99906	-14.999204
CH	-38.472138	-38.47566	-38.476653	-38.476839
NH	-55.219111	-55.223226	-55.224899	-55.225091
OH	-75.73433	-75.739939	-75.742386	-75.742667
HF	-100.449627	-100.456684	-100.460007	-100.460236
LiF	-107.423184	-107.43016	-107.432215	-107.432634
CN	-92.714928	-92.720175	-92.721855	-92.722082
CO	-113.311999	-113.318379	-113.320858	-113.321175
N2	-109.528046	-109.534502	-109.537125	-109.53752
NO	-129.899165	-129.905881	-129.9093058	-129.909686
O2	-150.338588	-150.345597	-150.349715	-150.350198
F2	-199.528356	-199.537481	-199.542052	-199.54265

We also plot the data with reference to the exact total energies from Ref. [1]. The QMC results are also shown for comparison and discussion.

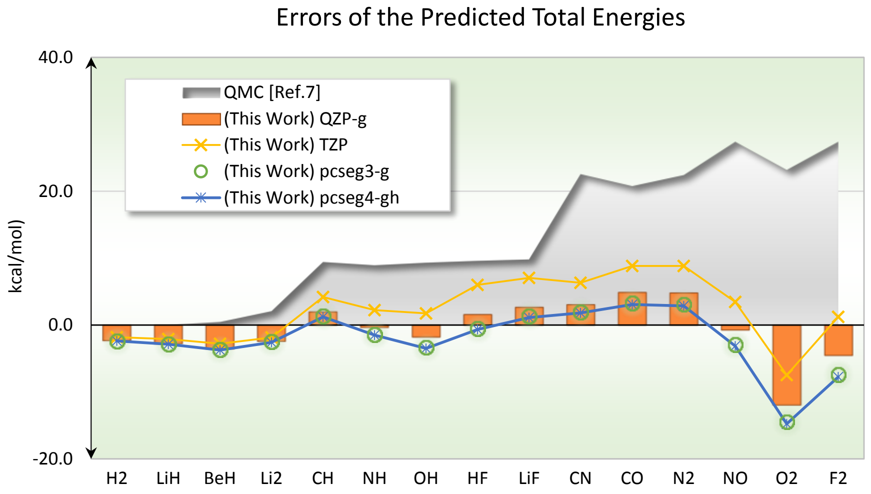

As seen from the graph, going from the TZP to QZP-g, the energies shift downward appreciably. But from QZP-g to pcseg3-g, the shift becomes small. Finally, going from pcseg3-g to pcseg4-gh, the shift is negligible. If we take the pcseg4-gh as the reference energies and see how much other basis sit above it, we get the following table.

Name	pcseg3 - pcseg4 [kcal/mol]	QZP - pcseg4 [kcal/mol]
H2	0.01	0.09
LiH	0.06	0.12
BeH	0.12	0.21
Li2	0.09	0.16
CH	0.12	0.74
NH	0.12	1.17
OH	0.18	1.71
HF	0.14	2.23
LiF	0.26	1.55
CN	0.14	1.20
CO	0.20	1.75
N2	0.25	1.89
NO	0.24	2.39
O2	0.30	2.89
F2	0.38	3.24
**COUNT**	15	15
**MAE**	0.2	1.4
**ME**	0.2	1.4

From the table, pcseg4-gh should be very close to the basis set limit. Assuming the pcseg4gh is the basis set limit, pcseg3-g basis set error is 0.2 kcal/mol on average. QZP-g sits 1.4 kcal/mol above the basis set limit on average.

In this test we use DFT energies from our exchange-correlation functional; but the 0.2 kcal/mol error of the pcseg3-g is very close to the errors associated with HF energies of the basis pc3 reported by F. Jensen [7]. In Table 3 of Ref. [7], the pc3's error is 0.43 mH or 0.27 kcal/mol. Therefore, we believe our convergence test result is reasonable.

Finally, we look at the MAE for each basis as follows.

Name	TZP - exact [kcal/mol]	QZP - exact [kcal/mol]	pcseg3 - exact [kcal/mol]	pcseg4 - exact [kcal/mol]
H2	-1.8	-2.3	-2.4	-2.4
LiH	-2.1	-2.7	-2.8	-2.9
BeH	-2.8	-3.5	-3.6	-3.7
Li2	-1.8	-2.4	-2.5	-2.6
CH	4.2	2.0	1.3	1.2
NH	2.3	-0.3	-1.4	-1.5
OH	1.7	-1.8	-3.3	-3.5
HF	6.0	1.6	-0.5	-0.7
LiF	7.0	2.7	1.4	1.1
CN	6.3	3.0	2.0	1.8
CO	8.8	4.8	3.3	3.1
N2	8.8	4.8	3.1	2.9
NO	3.5	-0.7	-2.9	-3.1
O2	-7.5	-11.9	-14.4	-14.7
F2	1.2	-4.5	-7.4	-7.7
**COUNT**	15	15	15	15
**MAE**	4.4	3.3	3.5	3.5
**ME**	2.3	-0.8	-2.0	-2.2

Notice that the MAE for QZP-g, pcseg3-g, pcseg4-gh are very close. There are 3.3, 3.5, and 3.5 respectively. Therefore, we conclude that if we use QZP-g basis set, the reported MAE should not be off by 0.2 kcal/mol from the basis set limit.

References

1. O'Neill, D.P.; Gill, P.M.W. Benchmark correlation energies for small molecules. *Mol. Phys.* **2005**, *103*, 763–766, doi:10.1080/00268970512331339323.

2. Nemec, N.; Towler, M.D.; Needs, R.J. Benchmark all-electron ab initio quantum Monte Carlo calculations for small molecules. *J. Chem. Phys.* **2010**, *132*, 034111, doi:10.1063/1.3288054.

3. Brown, M.D.; Trail, J.R.; López Ríos, P.; Needs, R.J. Energies of the first row atoms from quantum Monte Carlo. *J. Chem. Phys.* **2007**, *126*, 224110, doi:10.1063/1.2743972.

4. Ranasinghe, D.S.; Petersson, G.A. CCSD(T)/CBS atomic and molecular benchmarks for H through Ar. *J. Chem. Phys.* **2013**, *138*, 144104, doi:10.1063/1.4798707.

5. D.R. Lide, *CRC Handbook of Chemistry and Physics: A Ready-Reference of Chemical and Physical Data* (CRC press, Florida, 2005).

6. Jensen, F. Unifying General and Segmented Contracted Basis Sets. Segmented Polarization Consistent Basis Sets. *J. Chem. Theory Comput.* **2014**, *10*, 1074–1085, doi:10.1021/ct401026a.

7. Jensen, F. Estimating the Hartree—Fock limit from finite basis set calculations. *Theor. Chem. Acc.* **2005**, *113*, 267–273, doi:10.1007/s00214-005-0635-2.
